# Supplementary material for: Identification of a pro-angiogenic functional role for FSP1-positive fibroblast subtype in wound healing
Source: Nat Commun. 2019 Jul 9;10:3027. doi: 10.1038/s41467-019-10965-9 (PMC6617456; doi:10.1038/s41467-019-10965-9)
Supplement: Supplementary file 1 — Supplementary Information [file 41467_2019_10965_MOESM1_ESM.pdf]

Identification of a pro-angiogenic functional role for FSP1 positive fibroblast subtype in wound healing

Sarika Saraswati[1*], Stephanie MW Marrow[1], Lester A Watch[1], and Pampee P Young[1.2*]

1. Department of Pathology, Microbiology, and Immunology, Vanderbilt University Medical Center, Nashville, Tennessee, USA.
2. American Red Cross, National Headquarters, Washington, D.C

Vanderbilt University Medical Center

1161 21st Ave S.

MCN C2217

Nashville TN, USA 37232-2561

*Corresponding Authors: pampee.young@redcross.org; sarika.saraswati@vanderbilt.edu

Supplementary Figure 1.

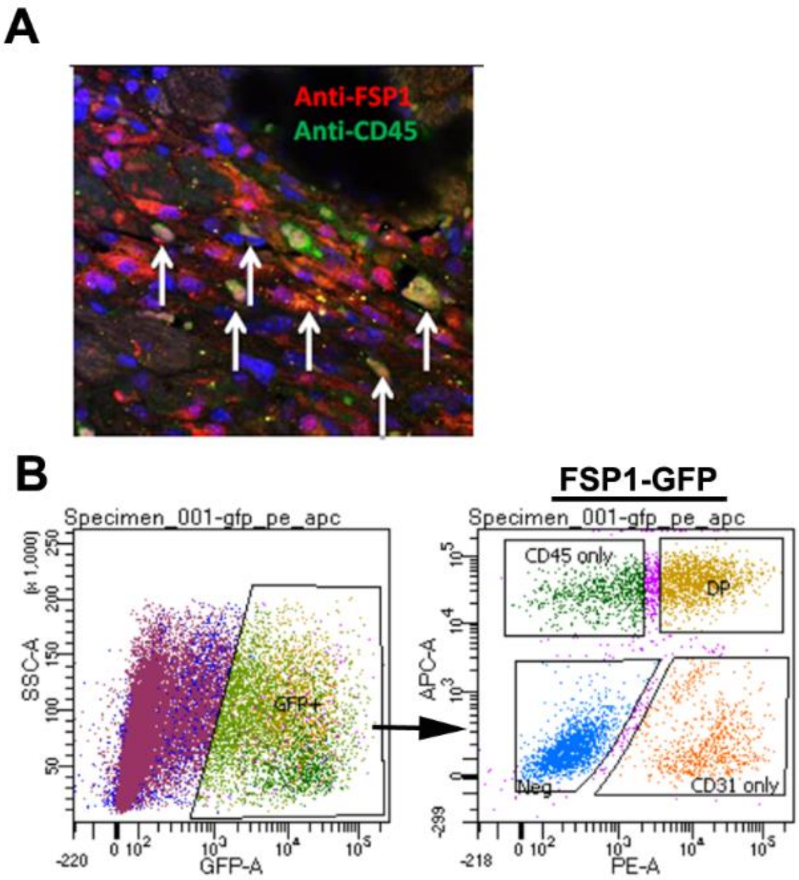

Supplementary Figure 1. FSP1 is expressed by endothelial, hematopoietic, as well as non-endothelial/hematopoietic cell populations post-infarct. A. Representative micrograph of the confocal analysis of FSP1 and CD45 co-immunostaining from murine heart 8 days post myocardial infarction (MI). FSP1 detected by Cy3 (red) fluorescence and CD45 by 488 (green). Arrow depicts the CD45 positive cells (hematopoietic cells) co-stained with FSP1 antibody. **B.** Representative dot plot of freshly isolated cells from FSP1-GFP mice left ventricle 10 days after MI. GFP^+ fibroblasts were gated as GFP^+/CD45-APC^+, GFP^+/CD31-PE^+, GFP^+/CD45-APC^+/CD31-PE^+ and GFP^+/CD45-APC^-/CD31-PE^- (n=3).

Supplementary Figure 2.

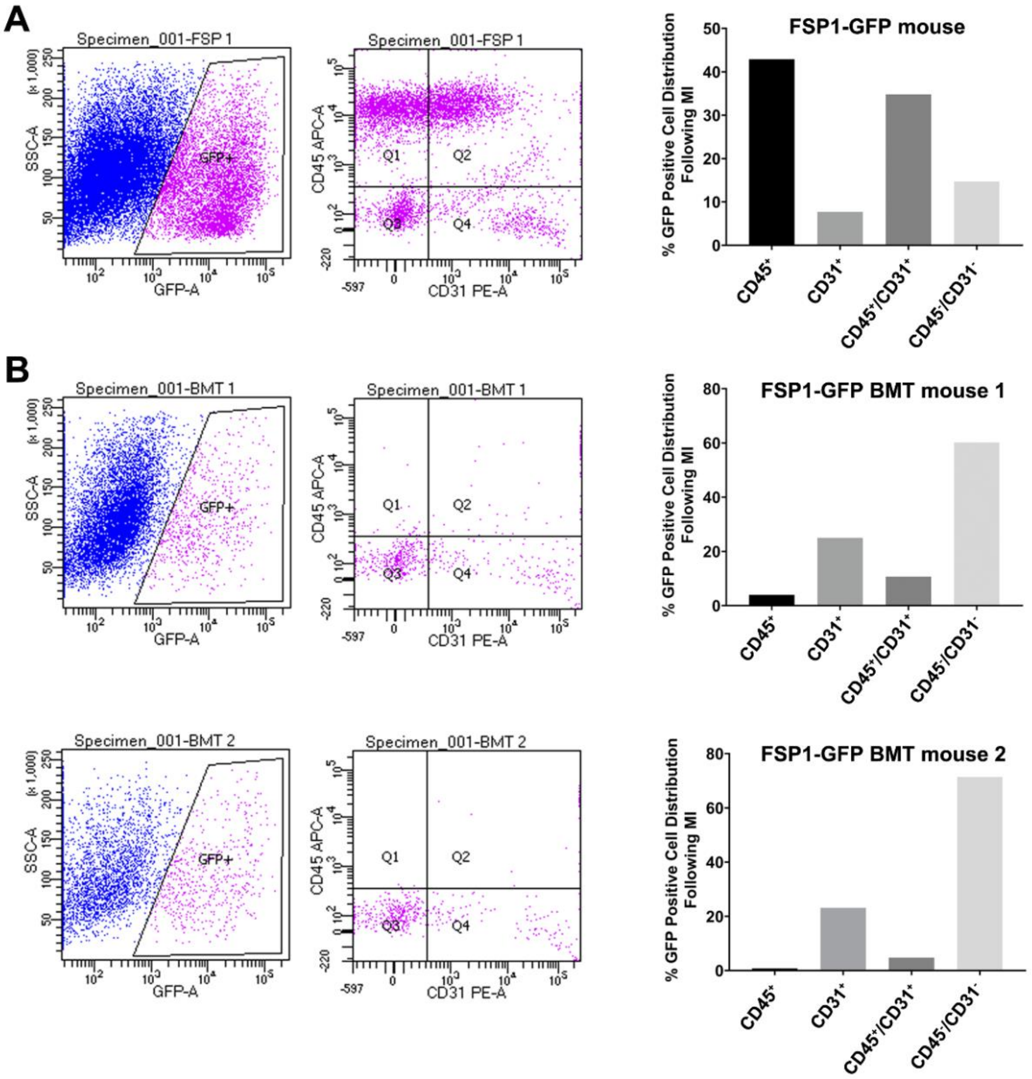

Supplementary Figure 2. Post-infarct cardiac FSP1⁺ fibroblasts are not recruited from the bone marrow but are activated at the site of injury. A (left panel). Flow cytometric evaluation of freshly isolated cells from FSP1-GFP mice left ventricle 10 days after MI represented by dot plot. **B (left panel).** Flow cytometric evaluation of freshly isolated cells from bone marrow transplanted (from C57Bl/6) FSP1-GFP mice left ventricle 10 days after MI represented by dot plot. GFP^+ cells were gated as GFP^+/CD45-APC^+, GFP^+/CD31-PE^+, and GFP^+/CD45-APC^-/CD31-PE^-. **A and B (right panel)**.

Quantification graphed as the % distribution of GFP^+ $CD45^{+/-}$ $CD31^{+/-}$ cells before (A) and after BMT (B).

Supplementary Figure 3.

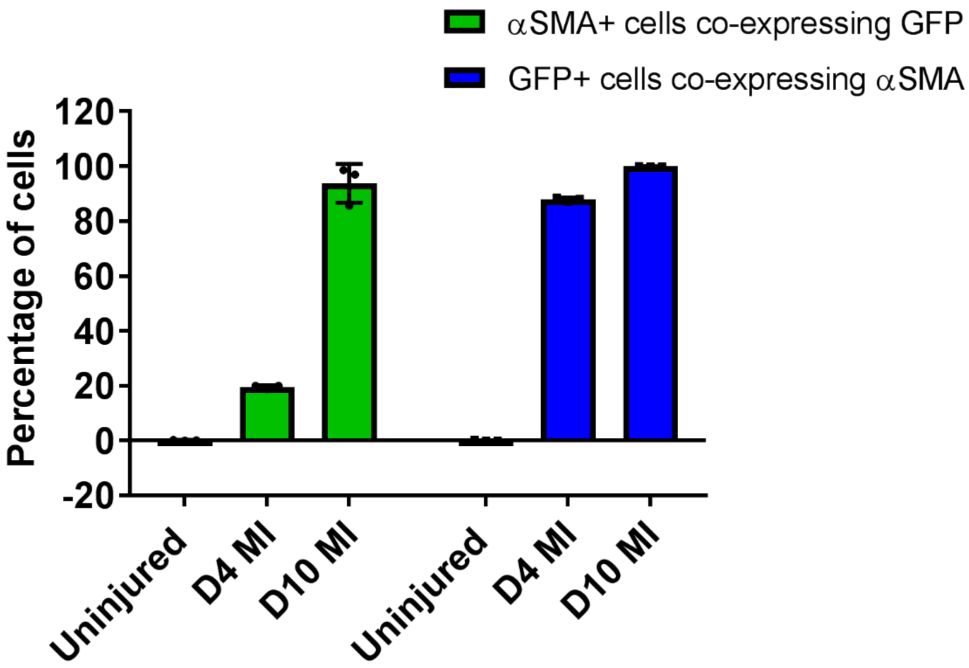

Supplementary Figure 3. Flow cytometric evaluation of freshly isolated cells from αSMA-GFP mice hearts representing % of αSMA expressing cells co-expressing GFP protein and GFP expressing cells co-expressing αSMA protein prior to MI and 4 and 10 days following MI (n=3).

Supplementary Figure 4.

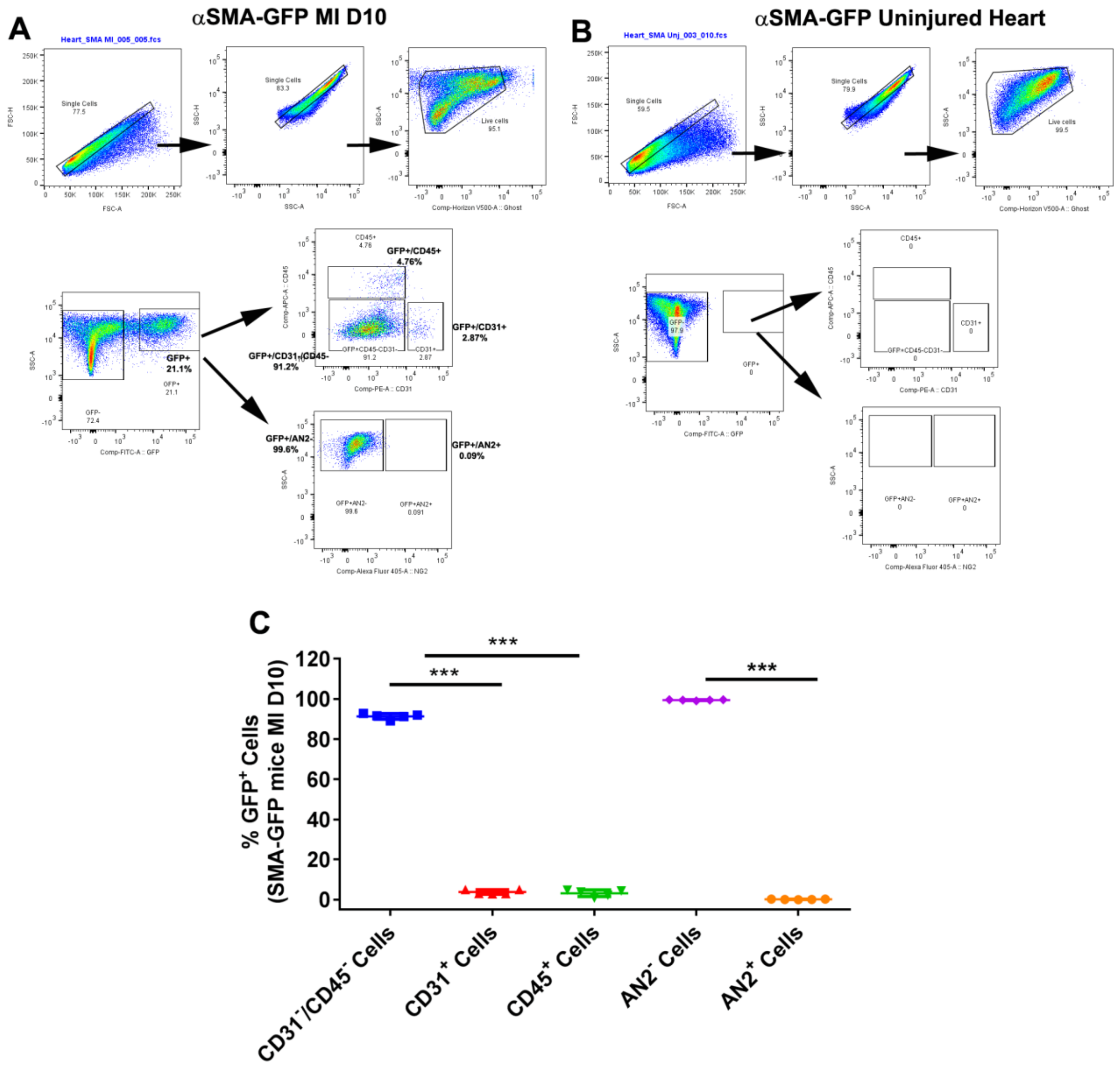

Supplementary Figure 4. (A, B, and C) Representative gating scheme of FACS analyses demonstrating %GFP+ live cells co-expressing CD31, CD45, or AN2 from αSMA-GFP mice uninjured hearts (B) or injured hearts (A) 10 days following myocardial infarction (MI). Atleast 5 separate isolations were done from individual mouse hearts.

The cells were freshly isolated and not pre-plated prior to analysis. The graphical quantification of the data is presented (C).

Supplementary Figure 5.

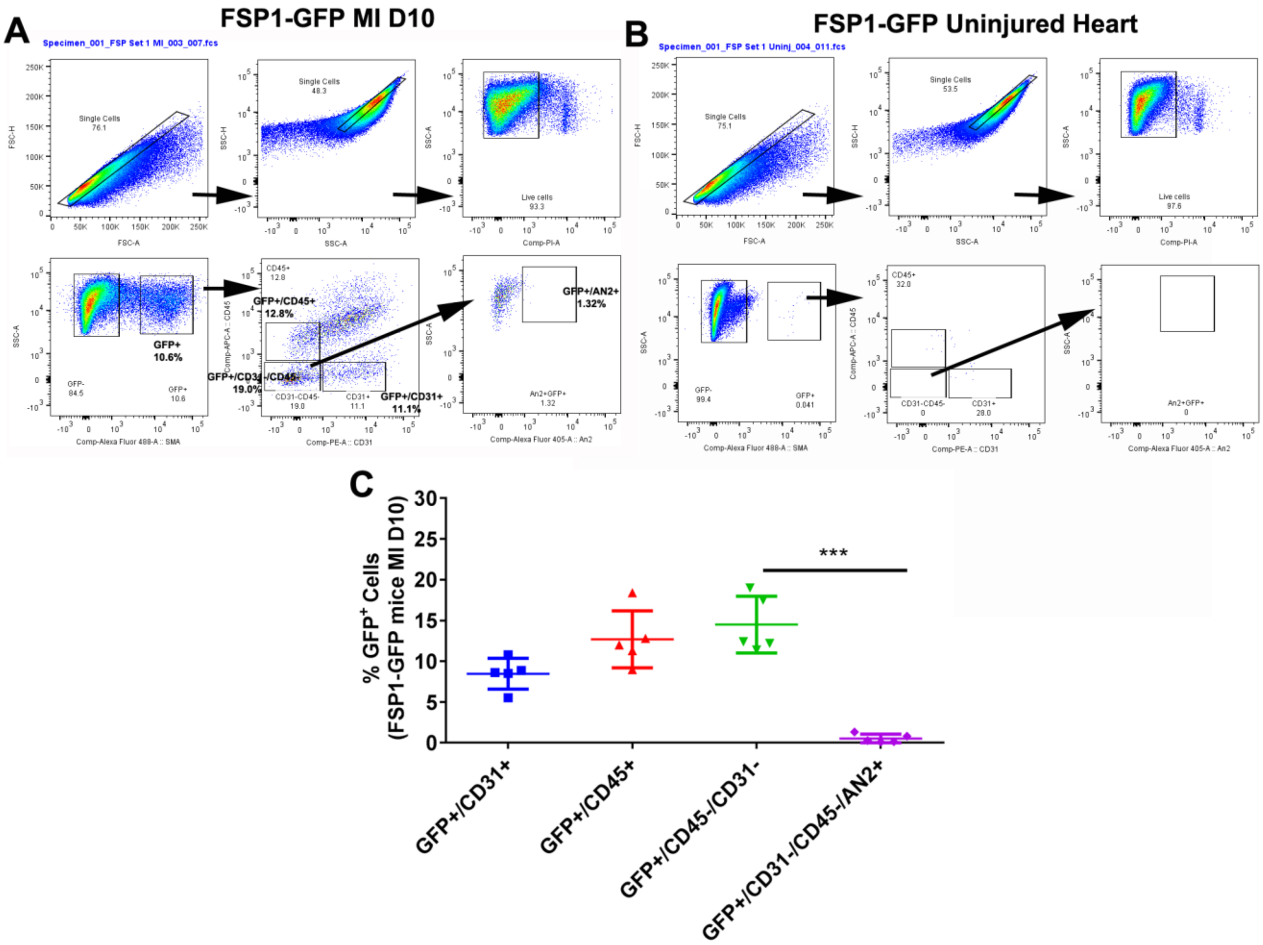

Supplementary Figure 5. (A, B, and C) Representative gating scheme of FACS analyses demonstrating %GFP+ live cells expressing CD31 or CD45 markers from FSP1-GFP mice uninjured hearts (B) or injured hearts (A) 10 days following myocardial infarction (MI). GFP+/CD31-/CD45- cells were then analyzed for the expression of pericyte marker AN2. Atleast 5 separate isolations were done from individual mouse

hearts. The cells were freshly isolated and not pre-plated prior to analysis. The graphical quantification of the data is presented (C).

Supplementary Figure 6.

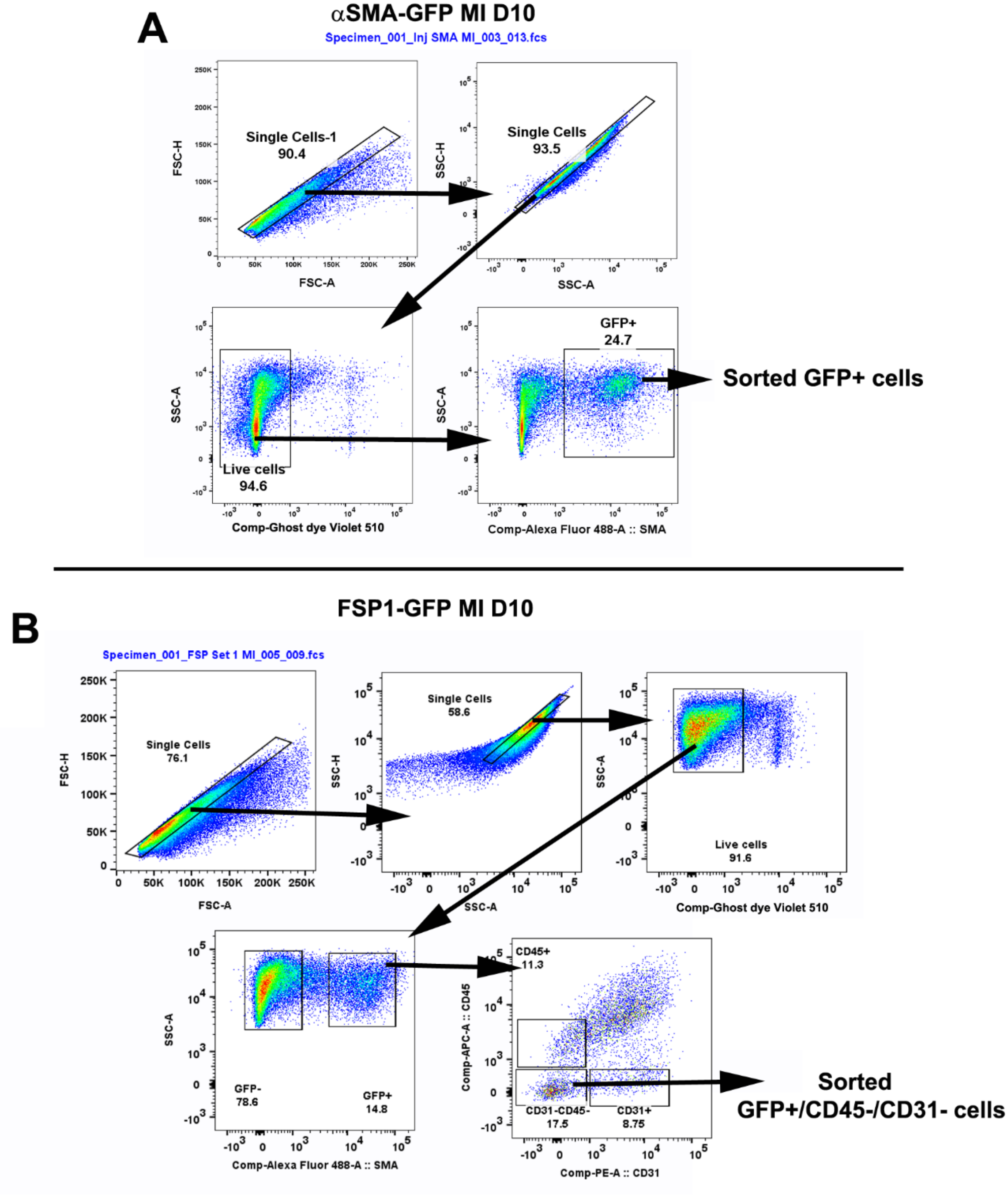

Supplementary Figure 6. (A and B) Gating strategy used for cell sorting. (A) Gating strategy to FACS sort GFP+ live cells from αSMA-GFP mice injured hearts10 days following myocardial infarction (MI). (B) Gating strategy to FACS sort GFP+/CD31-/CD45- live cells from FSP1-GFP mice injured hearts 10 days following MI. Atleast 5 separate isolations were done from 4-5 mouse hearts.

Supplementary Figure 7.

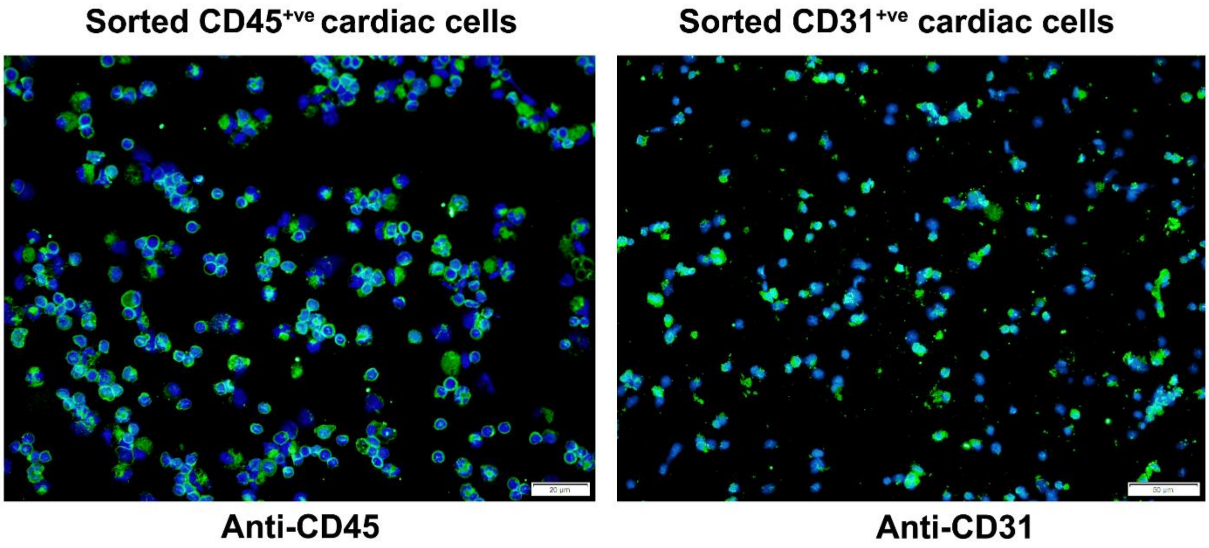

Supplementary Figure 7. Positive control for CD45 and CD31 immunostaining. Representative micrograph of immunostained cells detecting CD45 and CD31 by 488 (green) and DAPI (blue) for nuclear staining. Endothelial and hematopoietic cells (P0) were isolated from FSP1-GFP mice hearts 10 days after MI and cytospun on slides for immunostaining. (n=3).

Supplementary Figure 8.

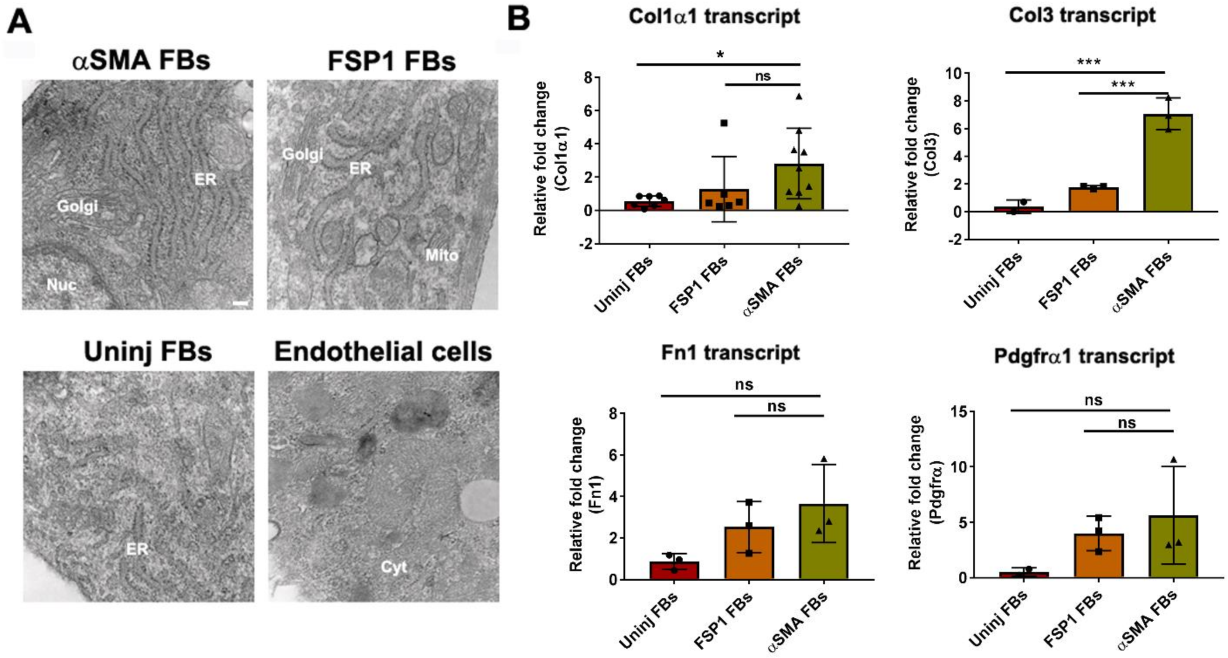

Supplementary Figure 8. Uninjured, FSP1, and aSMA FBs are ultrastructurally similar. A. FSP1 and αSMA expressing fibroblasts were isolated from mice 10 days following MI. Uninjured fibroblasts and endothelial cells were isolated from C57Bl/6 mice. Electron microscopy of αSMA, FSP1, and uninjured cardiac fibroblasts (FB), and endothelial cells (P0-P1) from mice. Scale bar=100 nM; 67000X magnification. Organelles including golgi apparatus (Golgi), mitochondria (Mito), rough endoplasmic reticulum (ER), cytoplasm (Cyt) and nucleus (Nuc) can be identified. **B**. Relative fold change of *Col1α1* (n=7 for uninjured, n=6 for FSP1$^+$, and n=9 for αSMA$^+$ fibroblasts), *Col3* (n=2 for uninjured, n=2 2for FSP1$^+$, and n=3 for αSMA$^+$ fibroblasts),, fibronectin (*Fn1*) (n=3 for uninjured, n=3 for FSP1$^+$, and n=3 for αSMA$^+$ fibroblasts), and *Pdgfrα1,* (n=2 for uninjured, n=3 for FSP1$^+$, and n=3 for αSMA$^+$ fibroblasts) transcripts measured by real

time RT-PCR in cultured uninjured, FSP1, and αSMA fibroblasts (P3-P5); $^{ns}p>0.05$, $*p <0.05$, and $***p <0.0001$ was calculated by one way ANOVA, n=3 experiments were performed; bar represent mean ±SD.

Supplementary Figure 9.

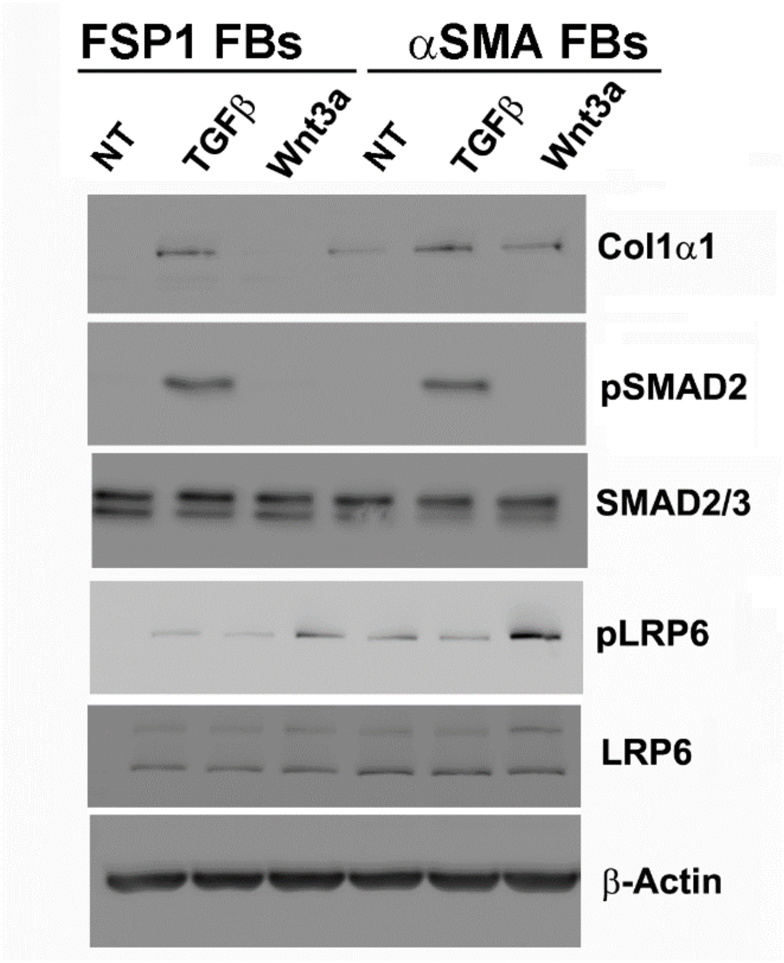

Supplementary Figure 9. FSP1⁺ and αSMA⁺ fibroblasts are responsive to pro-fibrotic cytokines. FSP1 and αSMA expressing fibroblasts were isolated from mice 10

days following MI. Cells were cultured and treated with treated with TGFβ or WNT3A proteins for 24 hours in 2% serum (P3-P5). Immunoblot analysis of COL1α1, pSMAD2, SMAD2, pLRP6, and LRP6 in FSP1 and αSMA fibroblast cell lysates. β-actin was used as loading control (n=3).

Supplementary Figure 10.

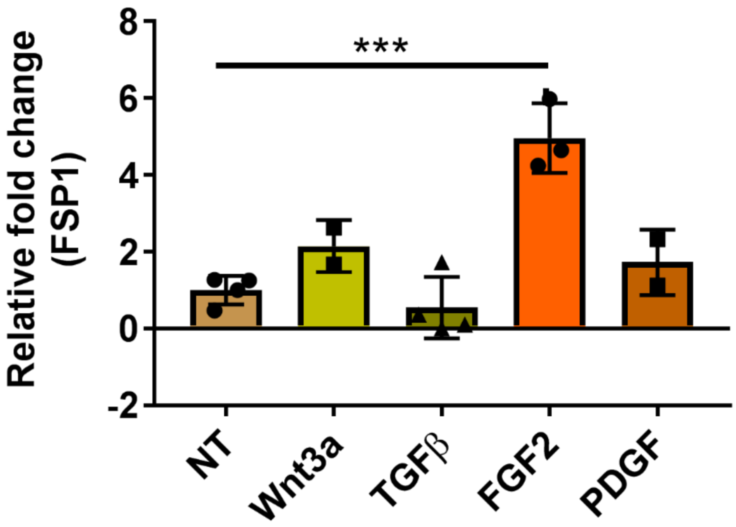

Supplementary Figure 10. Uninjured fibroblasts express FSP1 after treatment with different growth factors. Relative expression of *Fsp1* in uninjured fibroblasts (P3) untreated (n=4) and treated with WNT3A (50 ng/ml; n=2), TGFβ (10ng/ml; n=4), or FGF-2 (25 ng/ml; n=3), PDGFBB (1:1000; n=2) for 24 hours measured by semi-quantitative RT-PCR. ***p <0.0001 was calculated by one way ANOVA; bar represent mean ±SD.

Supplementary Figure 11.

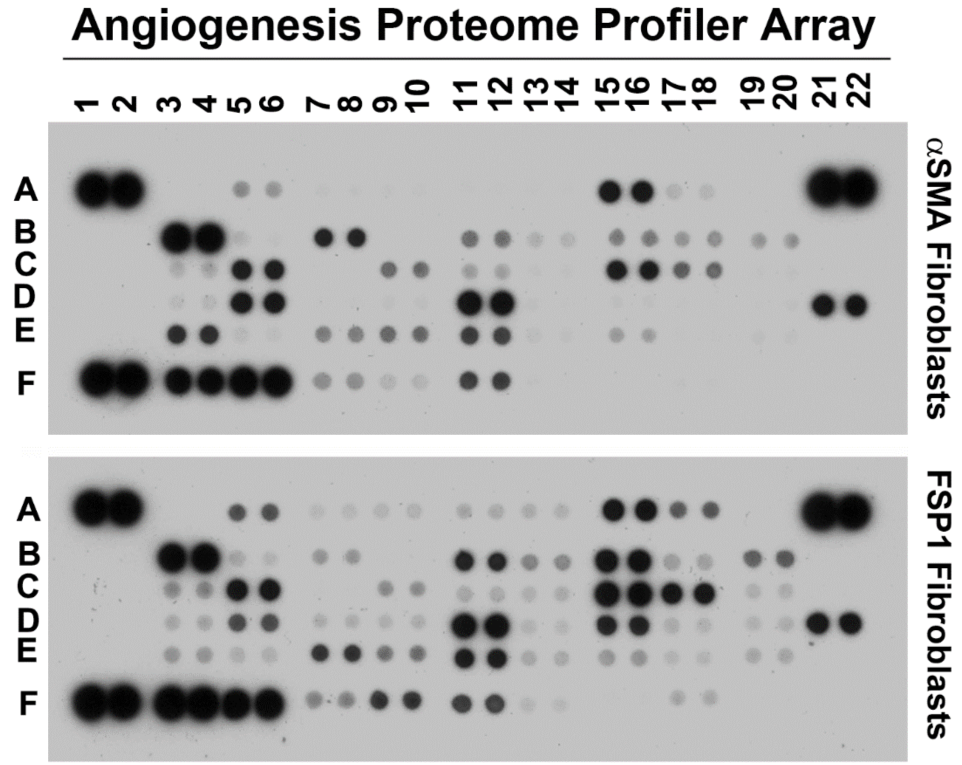

Supplementary Figure 11. Expression profile of angiogenesis-related proteins in FSP1 and αSMA fibroblasts. Immunoblot for angiogenesis-related proteins present in 300 µg of FSP1 and αSMA fibroblast cell lysates cultured for 72 hours (P3-P5) in 0.5% serum. Mouse angiogenesis proteome profiler array was used to perform the assay (n=2 technical replicates). Corresponding pixel density of each spot is provided in Supplementary Table 1.

Supplemenary Table 1.

Coordinate	Analyte/Control	FSP1 Lysate	αSMA Lysate
A1	Reference Spot	145869.5	147160
A2	Reference Spot	147185	144083
A3		16	14
A4		0	4
A5	ADAMTS1	16363	4705
A6	ADAMTS1	16726	2764
A7	Amphiregulin	3	15
A8	Amphiregulin	6	2
A9	Angiogenin	10	11
A10	Angiogenin	40	6
A11	Angiopoietin-1	560	3
A12	Angiopoietin-1	292	2
A13	Angiopoietin-3	181	2
A14	Angiopoietin-3	47	0
A15	Coagulation Factor III	46945	47004
A16	Coagulation Factor III	47437	46869
A17	CXCL16	13635	93
A18	CXCL16	14118	15
A19		66	4
A20		3	0
A21	Reference Spot	140365	148495
A22	Reference Spot	139409	149055
B1		44	0
B2		17	155
B3	Cyr61	99674	104713
B4	Cyr61	99376	98541
B5	DLL4	921	53
B6	DLL4	3	4
B7	DPPIV	2456	29696
B8	DPPIV	2452	29338
B9	EGF	2	0
B10	EGF	8	0
B11	Endoglin	33618	9835
B12	Endoglin	30026	9260
B13	Endostatin/Col XVIII	5970	104
B14	Endostatin/Col XVIII	5108	25
B15	Endothelin-1	57748	6261
B16	Endothelin-1	60339	6793
B17	FGF acidic	1956	2411

**B18**	**FGF acidic**	1438	3467
**B19**	**FGF basic**	13618	2037
**B20**	**FGF basic**	15808	2309
**B21**		35	0
**B22**		1	0
**C1**		355	0
**C2**		76	39
**C3**	**KGF**	6082	585
**C4**	**KGF**	7208	1297
**C5**	**Fractalkine**	48287	37949
**C6**	**Fractakine**	46467	36728
**C7**	**GM-CSF**	35	2
**C8**	**GM-CSF**	0	0
**C9**	**HB-EGF**	3901	9864
**C10**	**HB-EGF**	3482	9245
**C11**	**HGF**	545	1374
**C12**	**HGF**	393	986
**C13**	**IGFBP-1**	46	0
**C14**	**IGFBP-1**	208	1
**C15**	**IGFBP-2**	70810	37264
**C16**	**IGFBP-2**	66097	35377
**C17**	**IGFBP-3**	47282	12669
**C18**	**IGFBP-3**	44843	10439
**C19**	**IL-1a**	42	7
**C20**	**IL-1a**	77	5
**C21**	**IL-1b**	3	11
**C22**	**IL-1b**	0	0
**D1**		52	1
**D2**		0	0
**D3**	**IL-10**	511	40
**D4**	**IL-10**	807	55
**D5**	**IP-10**	19444	46360
**D6**	**IP-10**	19376	44902
**D7**	**KC**	180	14
**D8**	**KC**	112	98
**D9**	**Leptin**	36	1
**D10**	**Leptin**	109	4
**D11**	**MCP-1**	67828	62634
**D12**	**MCP-1**	66127	61391
**D13**	**MIP-1a**	33	3
**D14**	**MIP-1a**	27	5
**D15**	**MMP-3**	37937	13

**D16**	**MMP-3**	37605	15
**D17**	**MMP-8**	149	4
**D18**	**MMP-8**	54	2
**D19**	**MMP-9**	24	5
**D20**	**MMP-9**	90	138
**D21**	**NOV**	48329	49160
**D22**	**NOV**	48824	48128
**E1**		39	0
**E2**		0	28
**E3**	**Osteopontin**	3256	29137
**E4**	**Osteopontin**	2899	29398
**E5**	**PD-ECGF**	115	3
**E6**	**PD-ECGF**	76	4
**E7**	**PDGF-AA**	22759	6240
**E8**	**PDGF-AA**	23113	6338
**E9**	**PDGF-AB/PDGF-BB**	8401	10118
**E10**	**PDGF-AB/PDGF-BB**	8623	9960
**E11**	**Pentraxin-3**	37687	22722
**E12**	**Pentraxin-3**	37000	21366
**E13**	**Platelet Factor 4**	513	19
**E14**	**Platelet factor 4**	293	2
**E15**	**plGF-2**	2561	1605
**E16**	**PlGF-2**	3098	1140
**E17**	**Prolactin**	18	3
**E18**	**Prolactin**	29	7
**E19**	**Proliferin**	421	4
**E20**	**Proliferin**	516	24
**E21**		1	0
**E22**		0	0
**F1**	**Reference Spots**	122164	132011
**F2**	**Reference Spots**	110704	121897
**F3**	**SDF-1**	113140	85949
**F4**	**SDF-1**	112142.5	94331
**F5**	**Serpin E1**	85047	104152
**F6**	**Serpin E1**	95081	100574
**F7**	**Serpin F1**	5866	4868
**F8**	**Serpin F1**	6062	4478
**F9**	**Thrombospondin-2**	24931	63
**F10**	**Thrombospondin-2**	25168	6
**F11**	**TIMP-1**	24918	22589
**F12**	**TIMP-1**	24753	23302
**F13**	**TIMP-4**	8	4

**F14**	**TIMP-4**	-1	14
**F15**	**VEGF**	0	15
**F16**	**VEGF**	0	14
**F17**	**VEGF-B**	432	7
**F18**	**VEGF-B**	321	0
**F19**	**Negative Control**	0	0
**F20**	**Negative Control**	5	0

Supplementary Table 1. Pixel density of the immunoblot of angiogenesis-related proteins present in FSP1+ and αSMA+ fibroblast cell lysates identified by mouse angiogenesis proteome profiler array (n=2).

Supplementary Table 2.

Oligo Name	Forward (5'-3')	Reverse (5'-3')
Alpha SMA	CAGGCATGGATGGCATCAATCAC	ACTCTAGCTGTGAAGTCAGTGTCG
Angpt1-1	CACGTGGAGCCGGATTTCT	ATCTGGGCCATCTCCGACTT
Col1α1	GCCAGATGGGTCCCCGAGGT	GGGGGTCCAGCAGCACCAAC
Collagen 3	GAAAAAACCCTGCTCGGAATT	GGATCAACCCAGTATTCTCCACTCT
Fgf1-1	AGGAAACGTCCACAGTCAGG	CTCCTACGCCCACTCTTCAG
Fn-1	CGAGGTGACAGAGACCACAA	CTGGAGTCAAGCCAGACACA
Fsp1	CGGTTACCATGGCAAGACCC	TGTGCGAAGAAGCCAGAGTAAG
Gremlin	AGCAAAAGGGTTTTCCTGAT	AATGGTCAGCATTTCACCCT
AN2/NG2-1	CCTCAGAGCCCTATCTCCACGTAGC	CATCACCAAGTAGCCAGCGTTCG
PDGFRα	CAAACCCTGAGACCACAATG	TCCCCCAACAGTAACCCAAG
VEGF-A-2	AAAGGCTTCAGTGTGGTCTGAGAG	GGTTGGAACCGGCATCTTTATC
VEGF-B-2	TTAGAGCTCAACCCAGACACCTGTA	CCTGTGAAGCAGGGCCATAA
18S	CGCCGCTAGAGGTGAAATTCT	CGAACCTCCGACTTTCGTTCT

Supplementary Table 2. Primers used to analyze the gene expression changes by quantitative real time RTPCR analysis.

Supplementary Table 3. Tabular presentation of flow cytometry data at baseline and 10 days following myocardial injury in αSMA-GFP mice hearts.

Freshly isolated cardiac cells	GFP+ from live cells	GFP+/AN2+	GFP+/AN2-	GFP+/CD31+	GFP+/CD45+	GFP+/CD31-/CD45-
**αSMA-GFP MI (n=5)**	15.20%	0.24%	99.40%	2.98%	3.78%	91.60%
	8.79%	0.03%	99.40%	5.05%	4.35%	89.10%
	21.10%	0.09%	99.60%	2.87%	4.76%	91.20%
	15.30%	0.30%	99.10%	3.10%	2.18%	92.80%
	10.50%	0.11%	99.60%	4.98%	0.84%	92.00%
**Average**	14.18%	0.15%	99.42%	3.80%	3.18%	91.34%
**Std Dev**	0.0482	0.0011	0.0020	0.0112	0.0164	0.0138
**αSMA-GFP Uninjured heart (n=6)**	0.02%	0%	100%	11.10%	0%	88.90%
	0%	0%	0%	0%	0%	0%
	0%	0%	0%	0%	0%	0%
	0%	0%	0%	0%	0%	0%
	9.21E-05	0%	100%	25.00%	0%	75.00%
	0.03%	0%	100%	0%	30.00%	70.00%
**Average**	0.01%	0.00%	50.00%	6.02%	5.00%	38.98%
**Std Dev**	0.0001	0.0000	0.5477	0.1031	0.1225	0.4315

Supplementary Table 3. Flow cytometric evaluation of %GFP+ live cells present in the αSMA-GFP mice hearts at baseline (uninjured; n=6; bottom) or 10 days following myocardial infarction (MI; n=5; top). Percentage of GFP+ cells expressing markers of hematopoietic cells (CD45), endothelial cells (CD31) and pericytes/vascular smooth muscle cell marker (AN2) were also evaluated in both uninjured and injured hearts.

Supplementary Table 4. Tabular presentation of flow cytometry data at baseline and 10 days following myocardial injury in FSP1-GFP mice hearts.

Freshly isolated cardiac cells	GFP+ from live cells	GFP+/CD31+	GFP+/CD45+	GFP+/CD45-/CD31-	GFP+/CD31-/CD45-/AN2+
**FSP1-GFP MI (n=5)**	17.00%	5.04%	8.99%	12.20%	0.82%
	12.60%	8.29%	12.00%	12.40%	0.29%
	10.60%	11.10%	12.80%	19.00%	1.32%
	5.31%	7.72%	18.40%	11.40%	0.15%
	14.80%	8.75%	11.30%	17.50%	0.10%
**Average**	12.06%	8.18%	12.70%	14.50%	0.54%
**Std Dev**	0.0447	0.0218	0.0349	0.0348	0.0052
**FSP1-GFP Uninjured heart (n=5)**	0%	0%	0%	0%	0%
	0.02%	0%	0%	0%	0%
	0%	0%	0%	0%	0%
	0.33%	19.70%	18.00%	14.00%	0%
	0.04%	28.00%	32.00%	0%	0%
**Average**	0.08%	9.54%	10.00%	2.80%	0.00%
**Std Dev**	0.0014	0.1339	0.1456	0.0626	0.0000

Supplementary Table 4. Flow cytometric evaluation of %GFP+ live cells present in the FSP1-GFP mice hearts at baseline (uninjured; n=5; bottom) or 10 days following myocardial infarction (MI; n=5; top). Percentage of GFP+ cells expressing markers of hematopoietic cells (CD45) and endothelial cells (CD31) were also evaluated. Gated GFP+/CD45-/CD31- cells were analyzed for the presence of pericytes/vascular smooth muscle cell marker (AN2) in both uninjured and injured hearts.
