# Supplementary material for: Identification of a pro-angiogenic functional role for FSP1-positive fibroblast subtype in wound healing
Source: Nat Commun. 2019 Jul 9;10:3027. doi: 10.1038/s41467-019-10965-9 (PMC6617456; doi:10.1038/s41467-019-10965-9)
Supplement: Supplementary file 3 — Reporting summary [file 41467_2019_10965_MOESM3_ESM.pdf]

nature research

Corresponding author(s): Pampee P. Young and Sarika Saraswati

Last updated by author(s): May 29, 2019

Reporting Summary

Nature Research wishes to improve the reproducibility of the work that we publish. This form provides structure for consistency and transparency in reporting. For further information on Nature Research policies, see Authors & Referees and the Editorial Policy Checklist.

Statistics

For all statistical analyses, confirm that the following items are present in the figure legend, table legend, main text, or Methods section.

n/a	Confirmed	
☐	☒	The exact sample size (n) for each experimental group/condition, given as a discrete number and unit of measurement
☐	☒	A statement on whether measurements were taken from distinct samples or whether the same sample was measured repeatedly
☐	☒	The statistical test(s) used AND whether they are one- or two-sided<br>*Only common tests should be described solely by name; describe more complex techniques in the Methods section.*
☒	☐	A description of all covariates tested
☒	☐	A description of any assumptions or corrections, such as tests of normality and adjustment for multiple comparisons
☐	☒	A full description of the statistical parameters including central tendency (e.g. means) or other basic estimates (e.g. regression coefficient) AND variation (e.g. standard deviation) or associated estimates of uncertainty (e.g. confidence intervals)
☒	☐	For null hypothesis testing, the test statistic (e.g. F, t, r) with confidence intervals, effect sizes, degrees of freedom and P value noted<br>*Give P values as exact values whenever suitable.*
☒	☐	For Bayesian analysis, information on the choice of priors and Markov chain Monte Carlo settings
☒	☐	For hierarchical and complex designs, identification of the appropriate level for tests and full reporting of outcomes
☒	☐	Estimates of effect sizes (e.g. Cohen's d, Pearson's r), indicating how they were calculated

Our web collection on statistics for biologists contains articles on many of the points above.

Software and code

Policy information about availability of computer code

Data collection	CLC Genomic Workbench 8 (Qiagen)
Data analysis	Purified RNA was sent to Vanderbilt sequencing core (VANTAGE), paired end sequencing was performed at 75X depth. Raw data (FASTQ files) were uploaded to CLC Genomic Workbench 8 (Qiagen) and data quality assessment performed. Sequences were aligned to the Grmc38 platform of the Mus musculus genome using Tuxedo Suite, followed by normalization (RPKM). Data were annotated using Ensemble Release 83 and quantified at the gene level using CuffDiff. Since replicates were not use, proportional-based statistical analysis was performed using Baggerly's t-test with multiple hypothesis correction. Genes with fold-difference of 2 and p value < 0.05 were retained for functional analysis. DAVID bioinformatics Resource 6.7 platform was used for KEGG pathway analysis. The RNA-seq data are available at the NCBI GEO database with the accession number GSE130062.

For manuscripts utilizing custom algorithms or software that are central to the research but not yet described in published literature, software must be made available to editors/reviewers. We strongly encourage code deposition in a community repository (e.g. GitHub). See the Nature Research guidelines for submitting code & software for further information.

Data

Policy information about availability of data

All manuscripts must include a data availability statement. This statement should provide the following information, where applicable:

- Accession codes, unique identifiers, or web links for publicly available datasets
- A list of figures that have associated raw data
- A description of any restrictions on data availability

The RNA-seq data are available at the NCBI GEO database with the accession number GSE130062 (https://www.ncbi.nlm.nih.gov/geo/query/acc.cgi?acc=GSE130062). The data from the angiogenesis array is deposited on figshare and can be accessed at https://figshare.com/s/1e573d8d31707884f2b8. All other data supporting the finding of this study are available from the authors on request.

Field-specific reporting

Please select the one below that is the best fit for your research. If you are not sure, read the appropriate sections before making your selection.

☒ Life sciences ☐ Behavioural & social sciences ☐ Ecological, evolutionary & environmental sciences

For a reference copy of the document with all sections, see nature.com/documents/nr-reporting-summary-flat.pdf

Life sciences study design

All studies must disclose on these points even when the disclosure is negative.

Sample size	Where applicable power calculations were used to determine cohort size. No statistical methods were used to determine sample size animals from which cells were isolated for experimentation. The number of biological replicates are indicated in the figure legends.
Data exclusions	No data were excluded from the analysis.
Replication	All replication attempts were successful.
Randomization	No randomization done for these experiments
Blinding	In vitro and in vivo (sponge implantation) angiogenesis analyses were blinded.

Reporting for specific materials, systems and methods

We require information from authors about some types of materials, experimental systems and methods used in many studies. Here, indicate whether each material, system or method listed is relevant to your study. If you are not sure if a list item applies to your research, read the appropriate section before selecting a response.

Materials & experimental systems

n/a	Involved in the study	
☐	☒	Antibodies
☐	☒	Eukaryotic cell lines
☒	☐	Palaeontology
☐	☒	Animals and other organisms
☐	☒	Human research participants
☒	☐	Clinical data

Methods

n/a	Involved in the study	
☒	☐	ChIP-seq
☐	☒	Flow cytometry
☒	☐	MRI-based neuroimaging

Antibodies

Antibodies used	APC-CD45 (BD Bioscience; Clone: 30-F11; Cat No:559864, Lot No: C0451061118202), PE-CD31 (BD Bioscience; Clone MEC 13.3; Cat No:553373; Lot No: 6014987), Ghost DyeTM Violet 510 (Tonbo Biosciences; Cat No: 13-0870), purified anti-mouse CD16/ CD32 (Tonbo Biosciences; Clone No:2.4G2; Cat No:70-0161; Lot No:), anti-feeder cells-APC (MEF-SK4) (Miltenyi Biotec, Cat No: 130-102-900), Anti-AN2/NG2 (Miltenyi Biotec, Cat No:130-097-455), 7AAD (Molecular Probes; Cat No: A13010), anti-CD31/ platelet endothelial cell adhesion molecule-1 (PECAM-1; PharMingen; clone: MEC 13.3 (RUO); Cat No: 557355), Fibroblast Specific Protein 1 (FSP1) (Millipore; Cat No: 07-2274), α-Smooth Muscle Actin (αSMA) (Sigma, clone: 1A4; Cat No: A2547), COL1α1 (mdbioproducts; Cat No: 203002), Periostin (Santa Cruz; Cat No:SC67233), Vimentin (Sigma; Cat No:V2258), CD31 (BD Biosciences; Cat No: 553370), CD45 (BD Biosciences; Cat No: 553076), AN2/NG2 (Invitrogen; Cat No: MA5-24247), VWF Polyclonal Antibody (Thermo Fisher; Cat No: PA5-16634), VEGFR2 (Flk1) SINGLE CHAIN ANTIBODY (Fitzgerald; Cat No:10R-V106A), goat anti-mouse-Alexa Fluor 488 (Molecular Probes: A11029) or goat anti-rabbit-FITC (Southern Biotech; Cat No: 4050-02), goat anti-rat-Alexa Fluor 488 or Alexa Fluor 647 (Molecular Probes; Cat No: A11006 or; Thermo-Fisher; Cat No: A21247), or goat anti-chicken-Fluorescein (FITC) (Jackson Immunoresearch Laboratories; Cat No: 103-095-155), donkey anti-rat alexa fluor 647 (abcam: Cat No: ab175670), collagen type I (MD Bioproducts, Cat No:203002), pSMAD2 (cell signaling, Cat No:3108L), SMAD 2/3 (cell signaling, Cat No:5678S), LRP6 (Santa Cruz Biotechnology, Clone: C10; Cat No:SC-25317), pLRP6 (cell signaling, Cat No: 2568S) or β-actin (Sigma Aldrich, Cat No:A5441)
Validation	APC-CD45 (BD Bioscience; Clone: 30-F11; Cat No:559864), PE-CD31 (BD Bioscience; Clone MEC 13.3; Cat No:553373), Ghost DyeTM Violet 510 (Tonbo Biosciences; Cat No: 13-0870), 7AAD (Molecular Probes; Cat No: A13010), purified anti-mouse CD16/ CD32 (Tonbo Biosciences; Clone No:2.4G2; Cat No:70-0161; Lot No:),anti-feeder cells-APC (MEF-SK4) (Miltenyi Biotec, Cat No: 130-102-900), Anti-AN2/NG2 (Miltenyi Biotec, Cat No:130-097-455) antibodies are validated for flow cytometry analyses by the manufacturer.<br><br>anti-CD31/platelet endothelial cell adhesion molecule-1 (PECAM-1; PharMingen; clone: MEC 13.3 (RUO) has been validated for Immunohistochemistry by the manufacturer.

AN2/NG2 (Invitrogen; Cat No: MA5-24247) is validated by us for Immunofluorescence analysis.

α-Smooth Muscle Actin (αSMA) (Sigma, clone: 1A4; Cat No: A2547) is validated by us for Flow Cytometry analysis and by manufacturer for Immunofluorescence analysis.

Fibroblast Specific Protein 1 (FSP1) (Millipore; Cat No: 07-2274), Periostin (Santa Cruz; Cat No:SC67233), Vimentin (Sigma; Cat No:V2258), CD31 (BD Biosciences; Cat No: 553370), CD45 (BD Biosciences; Cat No: 553076), VWF Polyclonal Antibody (Thermo Fisher; Cat No: PA5-16634) and VEGFR2 (Flk1) SINGLE CHAIN ANTIBODY (Fitzgerald; Cat No:10R-V106A) antibodies are validated for Immunofluorescence analyses by the manufacturer and also by us.

COL1α1 (mdbioproducts; Cat No: 203002) has been validated by manufacturer as well as by us for Immunofluorescence and Western blot analysis.

pSMAD2 (cell signaling, Cat No:3108L), SMAD 2/3 (cell signaling, Cat No:5678S), LRP6 (Santa Cruz Biotechnology, Clone: C10; Cat No:SC-25317), pLRP6 (cell signaling, Cat No: 2568S) and β-actin (Sigma Aldrich, Cat No:A5441) antibodies are validated by the manufacturer.

All secondary antibodies conjugated with flourophores [goat anti-mouse-Alexa Fluor 488 (Molecular Probes: A11029) or goat anti-rabbit-FITC (Southern Biotech; Cat No: 4050-02) or goat anti-rabbit-Cy3 (Jackson Immunoresearch Laboratories, Cat No: 711-165-152) , goat anti-rat-Alexa Fluor 488 or Alexa Fluor 647 (Molecular Probes; Cat No: A11006 or; Thermo-Fisher; Cat No: A21247), or goat anti-chicken-Fluorescein (FITC) (Jackson Immunoresearch Laboratories; Cat No: 103-095-155), donkey anti-rat alexa fluor 647 (abcam: Cat No: ab175670)] are validated by the manufacturer.

Eukaryotic cell lines

Policy information about cell lines

Cell line source(s)	Human umbilical vein endothelial cells (HUVECs) were purchased from Lonza (C2519A; pooled donor), FSP1 and aSMA expressing fibroblasts were isolated from mouse hearts following myocardial infarction. These cells express GFP therefore they were sorted by GFP expression. CD31 and CD45 expressing cells were excluded from the sorting of FSP1 expressing fibroblasts.
Authentication	Each cell line and primary cells were stained for their spcific identification markars.
Mycoplasma contamination	Cell lines were not tested for mycoplasma contamination.
Commonly misidentified lines (See ICLAC register)	NA

Animals and other organisms

Policy information about studies involving animals; ARRIVE guidelines recommended for reporting animal research

Laboratory animals	WT, FSP1-GFP, aSMA-GFP transgenic mice on C57/BL6 background were used for the study. Equal number of male and female mice were used for cell isolation. Male mice were used for sponge transplantation.
Wild animals	NA
Field-collected samples	NA
Ethics oversight	The Vanderbilt University Institutional Animal Care and Use Committee approved the protocol (Protocol number: M1600076-00) for this study.

Note that full information on the approval of the study protocol must also be provided in the manuscript.

Human research participants

Policy information about studies involving human research participants

Population characteristics	Human heart tissues were collected from cadavers without MI as well as from cadavers who passed away within two weeks post-MI.
Recruitment	Informed consent was obtained with consent for autopsy to use the post mortem tissues for research in a de-identified manner.
Ethics oversight	Paraffin blocks of post mortem human heart tissue were obtained from the Vanderbilt Medical Center Tissue Repository via IRB approval (Vanderbilt IRB No: 130881).

Note that full information on the approval of the study protocol must also be provided in the manuscript.

Flow Cytometry

Plots

Confirm that:

☒ The axis labels state the marker and fluorochrome used (e.g. CD4-FITC).

☒ The axis scales are clearly visible. Include numbers along axes only for bottom left plot of group (a 'group' is an analysis of identical markers).

☒ All plots are contour plots with outliers or pseudocolor plots.

☒ A numerical value for number of cells or percentage (with statistics) is provided.

Methodology

Sample preparation	Uninjured C57Bl/6 mice were used to isolate uninjured fibroblasts whereas FSP1-GFP and αSMA-GFP mice were used to isolate and sort GFP expressing fibroblasts 10 days following MI. Mice were euthanized by overdose of isoflurane followed by cervical dislocation. Heart tissue was dissected to isolate ventricular tissue, which was then placed into Kreba-Henseleit (KHB; Sigma; K3753a) buffer with 2.9 mM CaCl2 and 24 mM NaHCO3 and quickly minced. The minced tissue was then transferred to a digestion cocktail of 0.25 mg/mL Liberase Blendzyme 3 (Roche Applied Science), 20 U/mL DNase I (Sigma Aldrich), 10 mmol/L HEPES (Invitrogen) and 0.1% sodium azide in HBSS and stirred at 37°C for 20 min. Cells collected after digestion were passed through 40 µm filter and centrifuged at 1500 400 × g rpm for 15 minutes. To remove red blood cells, cell pellet was resuspended in 1ml of Red Blood Cell lysis buffer and incubated for 1 minute. Following incubation, cells were washed with the KHB buffer and centrifuged at 400 × g1500 rpm for 15 minutes. Cells were resuspended in PBS with 5% FBS. For uninjured fibroblast isolation, cells were plated into 6-well dish in media for separation of fibroblasts by selective adhesion for 4 hours. Unattached and dead cells were washed with PBS and fresh media was added to the attached fibroblast for culture. For FSP1 and αSMA expressing fibroblasts isolation, GFP expressing cells were sorted by fluorescence assisted cell sorting (FACS).
Instrument	4-laser FACS Aria III
Software	FlowJo, LLC
Cell population abundance	FSP1 expressing fibroblasts: GFP+/CD45-/CD31-; 10-20%<br>aSMA expressing fibroblasts: GFP+; 15-20%<br>Following sorting using GFP, cells were immunostained with aSMA or FSP1 antibodies to ensure purity.
Gating strategy	Cells were gated based on their FSC/SSC profile. Dead cells were excluded by either using 7AAD or Ghost dyeTM violet 510 . Unstained uninjured fibroblasts were used to set the background signal in the GFP channel post compensation. A gate was drawn to capture all cells producing a larger voltage pulse area than the established background in the GFP channel. GFP+ FSP1 cells were gated with APC-CD45 and PE-CD31 antibodies to exclude FSP1 expressing endothelial and hematopoietic cells. For αSMA-GFP mice, live GFP positive cells were selected by gating for 7-AAD or Ghost dyeTM violet 510 negative, GFP positive cells.

☒ Tick this box to confirm that a figure exemplifying the gating strategy is provided in the Supplementary Information.
